# Tofacitinib as Rescue Therapy in Steroid-Refractory Acute Severe Ulcerative Colitis: A Retrospective Cohort Study with Observations on Cytomegalovirus Coinfection

**DOI:** 10.7759/cureus.111357

**Published:** 2026-06-23

**Authors:** Subhadeep Banerjee, Dawesh Yadav, Anand Gupta, Ujjwal Kumar, Sunit Shukla, Vinod Kumar, Anurag K Tiwari, Vinit M Kumar, Ajay Singla, Nitesh K Patel

**Affiliations:** 1 Gastroenterology and Hepatology, Institute of Medical Sciences, Banaras Hindu University, Varanasi, IND; 2 Gastroenterology, Institute of Medical Sciences, Banaras Hindu University, Varanasi, IND

**Keywords:** acute severe ulcerative colitis, cmv colitis, ibd, jak inhibitor, rescue therapy, steroid-refractory, tofacitinib

## Abstract

Background and aims: Acute severe ulcerative colitis (ASUC) is a severe and potentially life-threatening condition in which one-third of patients are refractory to intravenous corticosteroids. Established rescue therapies--infliximab and cyclosporine--are limited by cost, administration constraints, and toxicity. Tofacitinib, an oral Janus kinase (JAK) inhibitor, possesses pharmacokinetic and mechanistic properties that may be advantageous in the inpatient setting. This study was conducted to assess the short-term efficacy and safety of tofacitinib as rescue therapy in steroid-refractory ASUC and to identify clinical predictors of treatment failure.

Methods: We conducted a retrospective cohort analysis of all patients with ASUC admitted to our tertiary gastroenterology unit between March 2023 and August 2025. ASUC was defined per Truelove and Witts' criteria. Patients who failed to respond to intravenous hydrocortisone by Day 3 and who could not receive infliximab (due to cost or contraindication) were initiated on tofacitinib (10 mg thrice daily for 3 days, followed by 10 mg twice daily). The primary outcome was clinical response by Day 5-7, defined as a reduction in Mayo score of ≥3 points (≥30% from baseline) with an absolute rectal bleeding sub-score of 0-1. Secondary outcomes included non-response rate, adverse events, and the impact of colonic cytomegalovirus (CMV) DNA burden on treatment response.

Results: Ten consecutive patients with steroid-refractory ASUC received tofacitinib rescue therapy. The mean age was 29.6 ± 9.1 years; 60% were male. Seven patients (70%) achieved clinical response within 5-7 days. Three patients (30%) failed to respond and required alternative rescue therapy or surgical consultation. CMV DNA was detected in the colonic tissue of four patients; both patients with high viral loads (>50,000 copies/mL) failed to respond to tofacitinib despite concurrent antiviral therapy. Adverse events were minimal; one patient developed bicytopenia of uncertain attribution.

Conclusion: Tofacitinib demonstrated a 70% short-term clinical response rate in steroid-refractory ASUC, with an acceptable safety profile. Higher colonic CMV DNA appeared to be associated with a lower likelihood of clinical response; however, this observation requires validation with further research. Tofacitinib represents a viable, cost-effective rescue option, particularly in resource-limited settings or when conventional biologics are contraindicated. Prospective comparative studies are needed to define its optimal positioning within ASUC treatment algorithms.

## Introduction

ASUC is one of the most severe presentations of ulcerative colitis (UC), affecting approximately one-fifth to one-third of patients, while up to one-third of the patients present with ASUC on diagnosis [1]. Despite available medical management, ASUC is a highly comorbid condition and may potentially result in complications involving a colectomy.

Intravenous corticosteroids, established as an effective first-line treatment in the Truelove and Witts trial, continue to serve as the backbone of first-line therapy for ASUC management [2]. However, a large portion of patients do not respond effectively to steroid therapy and must be escalated to rescue therapy with biologics and/or surgery [3,4]. Timely identification of steroid non-responders is imperative because an additional delay after week one is independently associated with both an unfavourable prognosis and higher rates of colectomy [3,5].

Infliximab and cyclosporine are the currently accepted rescue options [1,6]. However, there are limitations in practical applications. Infliximab is expensive, requiring intravenous administration, and may lose some efficacy in patients with extensive systemic inflammation and hypoalbuminemia, as it may result in faster clearance of the drug compounds and loss in the fecal tract secondary to the altered mucosal integrity [7]. Cyclosporine, however, possesses limited therapeutic windows due to potential nephrotoxicity, neurotoxicity, hypertension, and electrolyte derangements [8]. Also, CMV co-infection--a known sequela of severe steroid-refractory UC--lowers the sensitivity to immunosuppression and has been linked with an increased rate of colectomy [9, 10], requiring routine CMV characterization as recommended by the present guidelines [9-11].

Tofacitinib is an oral small-molecule Janus kinase inhibitor that primarily targets JAK1 and JAK3 with limited activity on the inhibition of JAK2. Impeding these pathways can modulate several pro-inflammatory cytokines, including IL-2 through IL-21, and internal mediators such as IFN-α/γ [12]. While its role has not yet been specified under international guidelines governing steroid-refractory ASUC [7], data from various studies indicate its effectiveness as an alternative rescue [13-22]. This medicine is a more affordable oral alternative in resource-stressed countries like India, in which biologic availability may be constrained by economic factors [18-19].

Our goal is to assess not only immediate efficacy but also safety profiles, and further impact of colonic CMV burden on patients' response after initiation of treatment with tofacitinib within our treatment facility.

## Materials and methods

Study design and setting

The current study was a single-center, retrospective cohort study conducted at the Department of Gastroenterology, Institute of Medical Sciences, Banaras Hindu University, India. Medical records of patients admitted with ASUC between March 2023 and August 2025 were assessed.

Patient selection

Patients were diagnosed with ASUC as per Truelove and Witts criteria [2]. The response to intravenous corticosteroids was measured according to the Oxford criteria: persistent stool frequency >8/day or 3-8 stools/day, and C-reactive protein level >45 mg/L on day 3, were indicative of steroid failure and triggered rescue therapy [23]. Patients who did not respond to corticosteroids according to Oxford criteria [23] and had contraindications to infliximab (active or latent tuberculosis, uncontrolled systemic infection, heart failure (NYHA class III/IV), demyelinating disorders, untreated chronic hepatitis B infection, recent malignancy) [24] or could not afford infliximab therapy were included.

Exclusion criteria were: active or latent tuberculosis, present opportunistic infection other than CMV (unless adequately treated), previous use of a biologic agent, known hypersensitivity to tofacitinib, and pregnancy.

CMV assessment and categorization

Colonic mucosal biopsies obtained during unprepared flexible sigmoidoscopy were analyzed for CMV DNA using quantitative real-time polymerase chain reaction (qPCR) [9]. CMV viral burden was quantified as copies/mL according to the reporting standards of the institutional molecular microbiology laboratory.

For analysis, CMV burden was categorized a priori into: high CMV burden: >50,000 copies/mL; low CMV burden: 1-50,000 copies/mL; no detectable CMV DNA.

These predefined thresholds were used to evaluate the relationship between CMV burden and response to tofacitinib rescue therapy, based on prior evidence suggesting poorer outcomes with higher CMV burden in steroid-refractory ulcerative colitis [9,10].

Standard inpatient management protocol

All patients underwent baseline evaluation, including complete blood count, serum electrolytes, CRP, fecal calprotectin (FCP), erect X-ray abdomen, and unprepared flexible sigmoidoscopy with biopsy for histopathological examination. Stool assays for Clostridium difficile toxin (CDI) and colonic tissue CMV DNA quantification were performed in all patients. Patients with elevated CMV DNA in colonic tissue were treated concurrently with oral valganciclovir or intravenous ganciclovir as clinically indicated [9].

All patients received standard supportive care with intravenous fluid resuscitation, correction of electrolyte abnormalities, low molecular weight heparin (LMWH) for thromboprophylaxis, and nutritional support as appropriate with haematinic supplementation if indicated. Patients were treated with IV hydrocortisone 100mg q6hrly for management of ASUC.

Tofacitinib administration

Tofacitinib was started between Day 3 and 5 of hospital admission after evidence for steroid-refractory behavior was recorded and following exclusion of contraindications. The regimen of dosing was titrated as a high-intensity induction (10 mg three times a day for three days) with a 10 mg twice-daily schedule for continued induction therapy, in accordance with published guidelines [13,25].

Outcome assessment

The primary outcome was assessed between Day 5 and Day 7 after initiation of tofacitinib and was defined as clinical response based on the Partial Mayo score, consisting of stool frequency, rectal bleeding, and physician global assessment components, without inclusion of endoscopic reassessment [25]. Clinical response was a decrease of ≥3 points relative to baseline and ≥30% compared to baseline, and rectal bleeding subscore of 0-1 [25].

Clinical assessments such as stool frequency and rectal bleeding were recorded prospectively by the treating gastroenterology team for each day of hospitalization. Global assessment of physician evaluation was made by the consultant gastroenterologist managing inpatient care.

Baseline endoscopic severity was evaluated using the Ulcerative Colitis Endoscopic Index of Severity (UCEIS) [26]. All endoscopic examinations were performed by experienced gastroenterologists at the study center. To reduce interobserver variation, UCEIS scoring was conducted on standardized predetermined criteria, and final scores were determined following an interpretation of endoscopic images and other reports reviewed by two independent gastroenterologists. In cases of discrepancy, consensus agreement was reached prior to analysis.

Ethical considerations

The study protocol was reviewed and approved by the Institutional Ethics Committee, Institute of Medical Sciences, Banaras Hindu University, Varanasi, India (IEC Approval No. IMS/IEC/FEBRUARY/2026/1316, dated 26 February 2026). Ethical approval for retrospective review of patient records was obtained prior to data analysis.

Statistical analysis

Descriptive statistics were used to summarize clinical and demographic data. Continuous variables were expressed as mean ± standard deviation (SD) or medians with interquartile range (IQR), while categorical variables were reported as frequencies and percentages [27].

The small sample size limited the ability to perform inferential statistical analysis or adjust for potential confounders. Hence, observed differences between subgroups, including those based on CMV burden, should be interpreted as exploratory observations requiring validation in larger prospective cohorts.

## Results

Patient characteristics and disease severity at baseline

Ten steroid-refractory ASUC patients received tofacitinib rescue therapy during the study period. The mean age was 29.6 ± 9 years (range: 15-45 years), and six patients (60%) were male. The median duration of disease was 9 months (IQR: 3-24 months; range: 3-48 months). Most patients had extensive colonic involvement (E3, Montreal classification; n=7), while three had left-sided colitis (E2) [26]. All patients had prior exposure to 5-aminosalicylates; two had prior azathioprine exposure or documented intolerance. None had previously received biologic therapy (infliximab or vedolizumab). Baseline demographic, clinical, laboratory, and endoscopic characteristics are detailed in Table 1.

**Table 1:** Baseline Demographics, Severity Parameters, and Treatment Response of Individual Patients Abbreviations: 5-ASA = 5-aminosalicylates; AZA = azathioprine; CRP = C-reactive protein; FCP = fecal calprotectin; UCEIS = Ulcerative Colitis Endoscopic Index of Severity; CMV = cytomegalovirus; E2 = left-sided colitis; E3 = extensive colitis (Montreal classification).

Variable	P1	P2	P3	P4	P5	P6	P7	P8	P9	P10
Age (years)	28	30	45	19	28	28	30	43	15	30
Sex	F	M	M	F	M	M	F	M	F	M
Disease extent (Montreal)	E3	E2	E2	E3	E3	E3	E3	E3	E3	E3
Disease duration (months)	3	12	18	6	24	3	3	48	4	24
Prior treatment	5-ASA	5-ASA	5-ASA	5-ASA	5-ASA	5-ASA	5-ASA	5-ASA+AZA	5-ASA	5-ASA+AZA
Stools/day	15	9	10	12	10	20	10	12	10	15
CRP (mg/L)	64	35	101	107	38	82	54	122	108	31.7
Albumin (g/dL)	2.8	2.9	3.6	3.3	3.7	2.5	2.1	2.6	3.1	2.7
Hemoglobin (g/dL)	7.8	8.7	8.1	9.2	7.1	7.6	6.4	8	9.2	9.2
FCP (µg/g)	804	1078	1287	569	545	350	850	220	450	800
UCEIS score	7	7	8	7	6	7	5	5	6	6
CMV DNA (copies/mL)	–	–	–	–	–	350,200	55	–	476	56,509
Response to tofacitinib	Yes	Yes	No	Yes	Yes	No	Yes	Yes	Yes	No

All patients conformed to Truelove and Witts' criteria for ASUC [2]. There were 9 to 20 episodes per day of bloody stools. CRP levels ranged from 31.7 to 122 mg/L, serum albumin from 2.1 to 3.7 g/dL, and hemoglobin from 6.4 to 9.2 g/dL, indicating extensive systemic inflammation and disease burden. Average values of FCP ranged from 220 to 1,287 µg/g. In total, UCEIS scores ranged from 5 to 8, proving severe mucosal disease with endoscopic severity high in all patients. 

Treatment course and primary outcome

All patients were treated first-line with intravenous hydrocortisone 100 mg q6h [28]. The initiation of tofacitinib occurred from Day 3 to Day 5 after steroid non-response. Following the first three days of high-intensity induction (10 mg three times daily), the dosage was changed to 10 mg twice daily [13,25]. For 70% of the patients, clinical improvement was achieved between five and seven days of initiation of tofacitinib, with clinically significant and prolonged reduction of stool frequency, rectal bleeding, and inflammatory parameters. Three patients (30%) failed to respond to therapy and were referred for colectomy (Table 2).

**Table 2:** Clinical Outcomes Following Tofacitinib Rescue Therapy CMV=cytomegalovirus

Outcome Parameter	Value
Total patients enrolled	10
Clinical response by Day 5-7	7 (70%)
Non-response	3 (30%)
Time to clinical response	5-7 days
Patients with colonic CMV DNA detected	4 (40%)
Patients with high CMV load (>50,000 copies/mL)	2 (20%)
Clinical response in high-CMV patients	0% (0/2)
Adverse events	1 (bicytopenia)
Referred for infliximab/colectomy	3 (30%)

CMV co-infection and its impact on treatment response

Colonic tissue CMV DNA was detected in four patients. Two patients had high viral loads (>50,000 copies/mL; Patients 6 and 10), and two had low viral loads (Patients 7 and 9). Neither patient with high CMV DNA burden achieved clinical response to tofacitinib despite concurrent antiviral therapy. In contrast, both patients with low CMV loads and five of six patients with undetectable colonic CMV DNA responded favorably to tofacitinib, yielding an overall response rate of 87.5% (7/8) among patients without high CMV burden; the treatment response is presented in Table 3. 

**Table 3:** Impact of Colonic CMV DNA Burden on Response to Tofacitinib CMV=cytomegalovirus

CMV Category	N	Clinical Response Rate
High CMV (>50,000 copies/mL)	2	0% (0/2)
Low CMV (1–50,000 copies/mL)	2	100% (2/2)
No CMV detected	6	83.3% (5/6)
Total	10	70% (7/10)

Safety assessment and adverse event monitoring

Patients were monitored daily during hospitalization for the development of adverse events following initiation of tofacitinib therapy. Safety monitoring included serial clinical assessment and laboratory evaluation comprising complete blood counts, liver function tests, renal function tests, serum electrolytes, and inflammatory markers as clinically indicated. Particular attention was given to infectious complications, thromboembolic events, hematologic abnormalities, hepatotoxicity, and herpes zoster reactivation, based on the known safety profile of tofacitinib [17,25].

Short-term safety outcomes were assessed throughout the inpatient stay and until discharge or escalation to alternative rescue therapy/colectomy. Adverse events were identified through review of inpatient clinical records, laboratory parameters, medication charts, and consultant progress notes.

One patient developed bicytopenia characterized by anemia and leukopenia during therapy. Because this patient was concurrently receiving valganciclovir for CMV infection, a recognized cause of bone marrow suppression, causality attributable solely to tofacitinib could not be definitively established [9]. Leukopenia improved following discontinuation of tofacitinib, whereas anemia persisted and required additional evaluation. No episodes of venous thromboembolism, serious opportunistic infection, herpes zoster reactivation, major cardiovascular events, or hepatotoxicity were observed during the short-term follow-up period.

To minimize confounding from concurrent therapies, all concomitant medications, including antivirals, antibiotics, corticosteroids, and thromboprophylaxis, were systematically documented and reviewed during adverse event assessment.

## Discussion

In this retrospective cohort study, we detected a 70% initial clinical response to tofacitinib as rescue therapy in patients with steroid-refractory acute severe ulcerative colitis (ASUC) in a resource-limited tertiary care center. These results expand upon the evidence for tofacitinib as an effective and safe alternative modality for these patients.

 For tofacitinib in ASUC, the study by Berinstein et al. built a strong case that high-intensity dosing (10 mg three times daily) was able to prevent colectomy in two out of four patients with failure of standard rescue therapies [13]. Subsequently, Jena et al. reported a short-term response rate of 75% in initial observational studies, establishing a preliminary benchmark for tofacitinib’s effectiveness in these settings [14]. Steenholdt et al.’s systematic review of 148 patients in 15 articles also confirmed the potential utility of tofacitinib as a rescue treatment for ASUC [18]. Both the 10 mg twice- versus thrice-daily therapies were found to provide consistent colectomy-free survival and comparable results in this study.

Indian studies added useful background. Malakar et al. reported an impressive 87.5% response rate (7 out of 8 patients) [15], while Kotwani et al. reported that all four patients who were first-line rescue treated with tofacitinib avoided colectomy [16]. Our cohort fits very well with these findings and further supports the drug’s utility in low-resource settings.

The TACOS trial--the first prospective, double-blinded, placebo-controlled RCT of tofacitinib in ASUC, yielded a breakthrough. This study was conducted at a single Indian center with 104 patients randomized into groups who took tofacitinib (10 mg 3 times a day) plus corticosteroids or corticosteroids plus placebo for a period of seven days. By Day 7, there were improved clinical parameters in 83% of patients receiving tofacitinib, versus 59% in the control group. In addition, the rescue therapy requirement on Day 7 was also dramatically decreased in the tofacitinib arm, and the 90-day colectomy risk was markedly lower. These results not only validated tofacitinib’s benefits as an early steroid adjunct but also implied a synergistic effect from the JAK blockade that may restore steroid-responsive effects through modulation of IL-2 signalling and glucocorticoid receptor biology [20].

A new TRIUMPH study published in Canada added additional prospective evidence, which indicated that patients taking tofacitinib 10 mg twice daily had a Day 7 response rate of 58.3% in hospitalized patients with a history of biologic failure, comprising 33.3% of the population. The average time to response was only 2.4 days--less than the classic 4-5 days with infliximab or cyclosporine--a major benefit in ASUC, in which delayed response increases the risk of colectomy [21].

Extrapolated data from the multicenter GETAID cohort (n=55) also found tofacitinib to have favorable evidence for its effectiveness with a three-month colectomy-free survival rate of 79% in high-risk patients failing other therapy [19]. These uniform results in a range of populations and healthcare systems enhance the effectiveness of tofacitinib.

From a comparative perspective, the paradigm-shifting network meta-analysis of Huang et al., reviewing 21 studies with over 2,000 patients [29], determined tofacitinib to be the first-ranked therapy to reduce immediate colectomy rates over accelerated infliximab, standard infliximab, tacrolimus, and cyclosporine. This strong indirect evidence reinforces the strength of proof for tofacitinib as a potential primary rescue choice for steroid-refractory ASUC.

Safety, however, remains a concern in this critically ill patient population over the long term, although the available data appear reassuring. A systematic review by Mpakogiannis et al. involving 134 patients found the most common adverse event was infection (11.9%), followed by venous thromboembolism (0.7%) among these cases [17]. Our lower thromboembolism rates in the studies represent the usual practice of low molecular weight heparin (LMWH) prophylaxis, which was also the norm in our cohort. Dural venous sinus thrombosis in the tofacitinib arm involved a single (1.9%) patient in the TACOS trial [20]. There were only a few adverse events in our series, including a single example of bicytopenia, which may be due to co-administration with valganciclovir rather than tofacitinib.

Combining real-world data with the landmark TACOS randomized controlled trial [20] and the TRIUMPH [21] prospective cohort enhances the credibility of tofacitinib's role in ASUC. Its high rating in Huang et al.'s network meta-analysis--superior even to infliximab in reducing colectomy--further suggests it can be considered as a first-line rescue strategy in appropriate patient populations [29].

Among several, the most novel and clinically relevant finding from our study was the strong association between increased colonic CMV DNA load and treatment failure. Patients with viral loads greater than 50,000 copies/mL had no response to tofacitinib despite being treated with antiviral therapy, compared to an 87.5% response in patients with low CMV burden. This was consistent with findings from published literature indicating that CMV disease diminishes the response to immunosuppressive therapies in steroid-refractory UC [10], and consistent with ECCO guidelines that support the evaluation of CMV in a consistent manner among all ASUC patients [9]. CMV can maintain mucosal inflammation through immune escape mechanisms that JAK inhibition alone cannot reverse at the mechanistic level [30]. Clinically, this may indicate that high levels of CMV DNA represent a stronger predictive source for poor response to tofacitinib and necessitate early surgical consideration and other therapeutic alternatives even when antiviral therapy is administered in the setting of patients with CMV.

Strengths and limitations

Strengths

This study offers accurate Indian data in a cohort described in comprehensive clinical, laboratory, and endoscopic detail. The use of objective severity indices (UCEIS, Mayo score) and systematic CMV quantification ensures methodological rigor when characterizing the cohort and identifying predictors of response. Our cohort, although small, adds to the limited Indian real-world data on tofacitinib use in steroid-refractory ASUC and further supports its utility in resource-limited settings.

Limitations

As this is a retrospective, single-center study design, generalization becomes difficult, and the small sample size does not allow multivariable analysis. The lack of a comparison arm precludes comparison against infliximab or cyclosporine. Long-term outcomes--including stable remission or delayed adverse events, and colectomy-free survival beyond the acute phase--could not be evaluated due to limited structured follow-up. The absence of systematic reassessment of CMV clearance in the acute aftermath of antiviral therapy could have affected response assessment in the CMV-positive group. Observations regarding the apparent association between higher colonic CMV DNA burden and poor response to tofacitinib should be interpreted cautiously and require validation in larger prospective cohorts. Although limited, this patient cohort adds clinically relevant, hypothesis-forming material in a population with few available therapeutic strategies.

## Conclusions

Tofacitinib demonstrated a 70% short-term clinical response rate as rescue therapy in steroid-refractory ASUC in this retrospective cohort, with a favorable safety profile during inpatient follow-up. High colonic CMV DNA load was strongly associated with tofacitinib failure, highlighting the critical need for systematic and quantitative CMV evaluation before initiating rescue therapy. Given its oral administration, rapid onset of action, predictable pharmacokinetics, absence of requirement for therapeutic drug monitoring, and relative cost-effectiveness compared to biologic agents, tofacitinib represents a practical and viable rescue option--particularly in resource-limited settings or when infliximab is contraindicated or unavailable.

Larger, prospective, comparative studies are required to formally define patient selection criteria, optimal dosing strategies, predictive biomarkers for response, and long-term outcomes, and to integrate tofacitinib into established international ASUC treatment algorithms.
